# Unveiling the molecular structure and role of RBBP4/7: implications for epigenetic regulation and cancer research

**DOI:** 10.3389/fmolb.2023.1276612

**Published:** 2023-11-13

**Authors:** Lize Cai, Bin Liu, Yufei Cao, Ting Sun, Yanyan Li

**Affiliations:** ^1^ The First Affiliated Hospital of Soochow University, Suzhou University, Suzhou, China; ^2^ Department of Neurosurgery, Qinghai Provincial People’s Hospital, Xining, China

**Keywords:** RBBP4, RBBP7, epigenetic complex, scaffold protein, cancer metabolism, targeted therapy

## Abstract

Retinoblastoma-binding protein (RBBP) family is a class of proteins that can interact with tumor suppressor retinoblastoma protein (pRb). RBBP4 and RBBP7 are the only pair of homologous proteins in this family, serving as scaffold proteins whose main function is to offer a platform to indirectly connect two proteins. This characteristic allows them to extensively participate in the binding of various proteins and epigenetic complexes, indirectly influencing the function of effector proteins. As a result, they are often highlighted in organism activities involving active epigenetic modifications, such as embryonic development and cancer activation. In this review, we summarize the structural characteristics of RBBP4/7, the complexes they are involved in, their roles in embryonic development and cancer, as well as potential future research directions, which we hope to inspire the field of epigenetic research in the future.

## Background

Retinoblastoma gene (*Rb*), the first discovered tumor suppressor gene, encodes retinoblastoma protein (pRb) that plays an important role in cell biological activities through unique epigenetic regulation processes (Mandigo et al., 2022; Zhou et al., 2022). These processes include ribosome mobilization, histone modifications, DNA modifications, non-coding RNA regulation, and so on. Therefore, once *Rb* is missing or mutated, the resulting deletion or mutation of the associated protein can cause a variety of cancers, such as retinoblastoma, osteosarcoma, adenocarcinomas, small cell lung cancer, breast cancer, and prostate cancer (Harbour and Dean, 2000; Guzman et al., 2020). As an epigenetic regulator, pRb often interacts with various proteins and complexes. The retinoblastoma-binding protein (RBBP) family has attracted attention in the early stages of exploration owing to its diverse epigenetic functions.

RBBP family contains a specific domain that interact with pRb. The first two isolated RBBP proteins, RBBP1 and RBBP2, were identified as they can bind to a region of pRb, similar to some transforming proteins produced by oncoviruses such as HPV or SV40 (Defeo-Jones et al., 1991). So far, seven major RBBP proteins (RBBP1/2/4/6/7/8/9) have been found be associated with diseases and have been extensively studied. It has been recognized that these RBBP proteins, in addition to sharing a binding domain that can interact with pRb, each contain many unique domains that can form complexes with other proteins and thus generate various biological effects (Wu et al., 2013; Li et al., 2018; Nabeel-Shah et al., 2021). As a result, these RBBP proteins have many different names depending on the focus of their different domains. For example, RBBP1 is also known as ARID1A (AT-rich interaction domain 1A), RBBP2 is also known as KDM5A (Lysine-specific demethylase 5A) or JARID1A (Jumonji/ARID domain-containing protein 1A), and RBBP6 is also known as PACT (p53-associated cellular protein-testes derived). The significant structural differences between these RBBP proteins suggest that their amino acid sequences are not highly homologous, which means that they cannot be strictly classified as a protein family. Specially, RBBP4 and RBBP7 are the only homologous pair of these proteins, which have highly similar protein sequences but also each contain unique functions (Huang et al., 1991; Qian et al., 1993). This review focuses on the molecular structural characteristics of RBBP4/7 and its role in epigenetic fields. It also summarizes the advancements in RBBP4/7 in the field of cancer and provides some insights for future targeted research based on these findings.

## The basic molecular features of RBBP4/7

**FIGURE 1:**
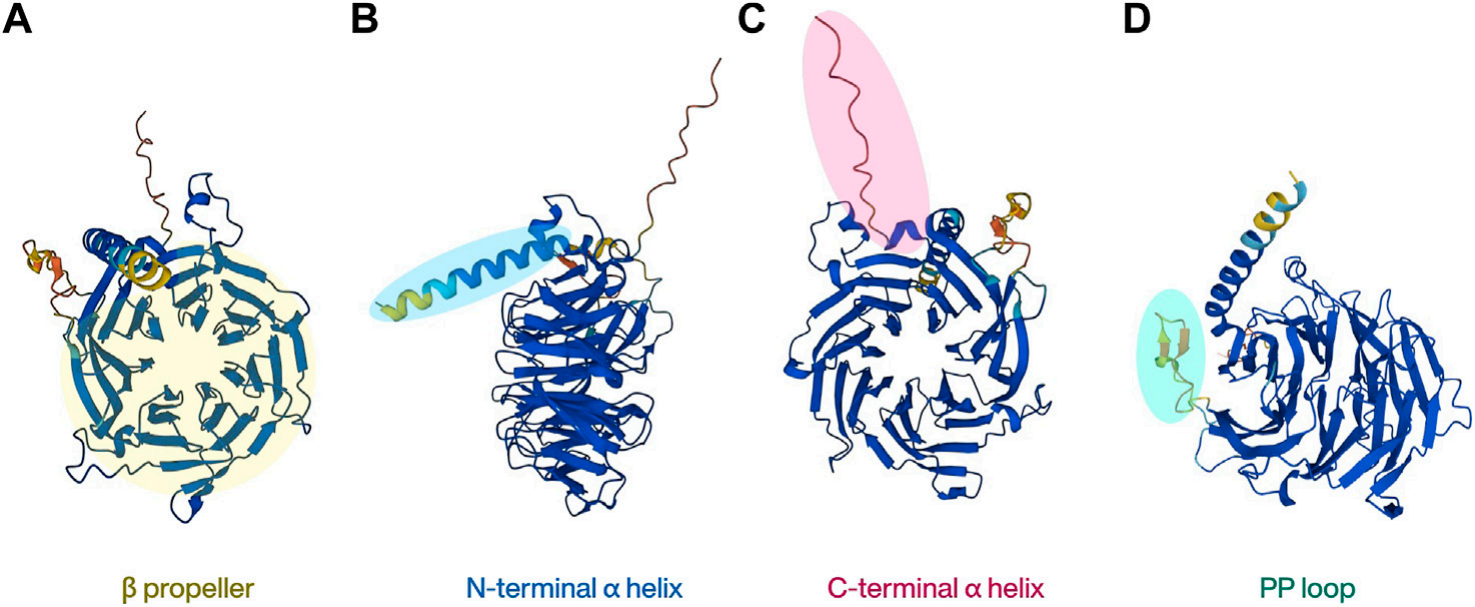
The basic structure of RBBP4. The highly homologous RBBP7 protein shares similar structural features.

The human *RBBP4* gene is located on chromosome 1p35 and encodes a polypeptide of 425 amino acids, whereas the human *RBBP7* gene is located on chromosome Xp22 and encodes a polypeptide of 469 amino acids. They share up to 92% sequence identity (Xu and Min, 2011). RBBP4 and RBBP7 were originally named as retinoblastoma associated protein 48 (RbAp48) and RbAp46, which also represent their respective molecular weights (Verreault et al., 1998). RBBP4/7 are both WD40 domain proteins and do not have any catalytic function (Hart et al., 2021). The WD40 protein family is a large protein superfamily in the human proteome that contains over 250 proteins. Its main characteristic, the highly conserved WD40 repeat (WDR) domain, is the most functionally diverse domain discovered, and over a thousand WDR domains have been annotated currently (Schapira et al., 2017; Ma et al., 2019). A typical WDR domain contains a β-propeller shaped like seven blades located at the center of the protein. This unique shape makes it look like a “donut” with a hole in the center, and other proteins or short peptides can interact with the top, bottom, or side of the donut, but not with the central channel. This property allows it to act as a platform for protein-protein interactions (PPI) and to build a network of protein actions as scaffolds (Stirnimann et al., 2010). RBBP4 and RBBP7 belong to the WDR RBBP4/RBBP7/MSI1 subfamily and highly homologous, so the conformations of the two proteins are very similar, comprising mainly of two α helices at the N/C terminal separately (Figures 1B, C) and a typical β-propeller (Figure 1A) with seven blades located in the middle (Murzina et al., 2008; Lejon et al., 2011). Another member of the protein family, MSI1, differs from the other two because it does not bind to pRb. Instead, it is better known for maintaining stem cell function, particularly in neural stem cells, by binding to RNA (Kameda-Smith et al., 2022; Chen et al., 2023).

As a scaffold protein, RBBP4/7 usually binds to other proteins to form a complex, that then interacts with histones, allowing it to exert diverse chromatin-related functions. In general, the N-terminal peptide of histone H3 binds to the central propeller of RBBP4/7. But uniquely, RBBP4/7 has a loop comprising of 18 amino residues, referred as PP loop, inserted at the sixth blade of the β-propeller and forms a negatively charged pocket with the N-terminal α-helix (Figure 1D). Histone H3/4 can also be recognized by RBBP4/7 by interacting with this pocket (Murzina et al., 2008). The interface of RBBP4/7 the histone binds to depends on the protein that has been fused to them (Lejon et al., 2011; Schmitges et al., 2011). For non-histone proteins, previous studies have found that the negative charge in the center of RBBP4/7 particularly tends to bind to target proteins with positively charged Arg-Arg-Lys residues (RRK motif) (Liu et al., 2018; Price et al., 2022). However, proteins without RRK motifs but with high density lysine and arginine residues can also bind to RBBP4/7 (Todd and Picketts, 2012). Therefore, non-histone proteins’ binding to RBBP4/7 does not rely on specific amino acid sequences, but more on the positive charge on the surface of the associated protein attracting with the negative charge in the center of RBBP4/7. This property is also the basis for the ability of RBBP4/7 to involve in PPI (Price et al., 2022).

## RBBP4/7 as a subunit in complex

As a scaffold protein, RBBP4/7 functions almost exclusively within protein complexes, and its binding partners and biological effects are highly dependent on the complex it is part of. The following are several protein complexes where RBBP4/7 is commonly found and their known functions:

### RBBP4/7 in CAF1

**FIGURE 2:**
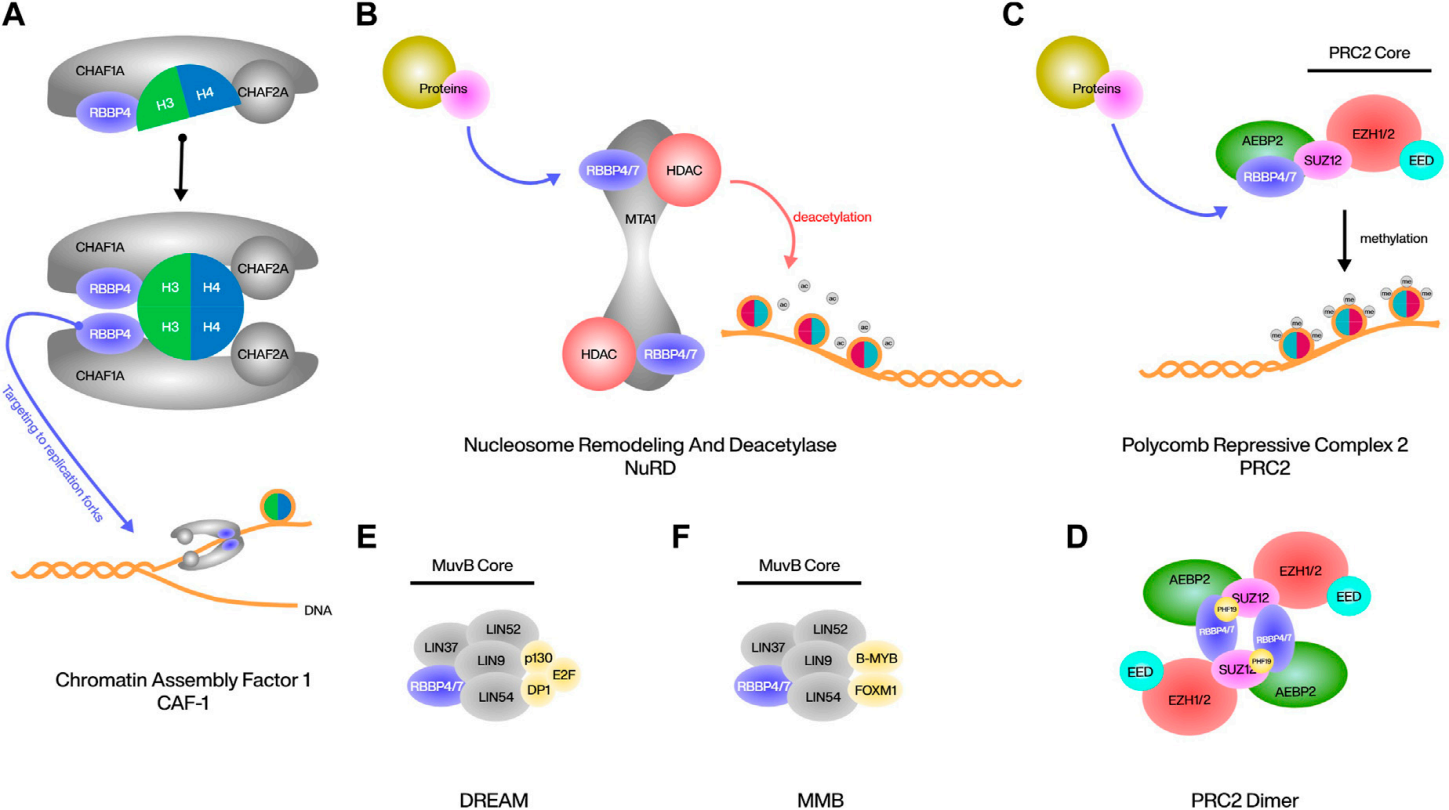
Various protein complexes involved in RBBP4/7.

The human chromatin assembly factor 1 (CAF1) complex is composed of three subunits, CHAF1A, CHAF1B and the smallest RBBP4. CAF1 acts as a histone chaperone to deposit newly synthesized histone H3/4 onto the DNA behind the replication fork, in which RBBP4 is responsible for the interaction of this complex with histones (Yu et al., 2015). Although the mode of binding of RBBP4 to histones has been mentioned above, it has been reported that in CAF1, RBBP4 binds dimer of histone H3/4 rather than monomer or tetramer, and RBBP4 can simultaneously interact with both H3 and H4 and induce structural rearrangements in H3-H4 during binding (Sauer et al., 2018). In addition to interacting with histones, RBBP4 is responsible for targeting CAF1 to replication forks at DNA breaks, and deletion of RBBP4 prevents DNA breaks from packaging to nucleosomes (Kadyrova et al., 2013) (Figure 2A). RBBP4 is also involved in the DNA repair process by CAF1, possibly by interacting with PCNA, but further studies are needed (Hengstschläger et al., 2003).

### RBBP4/7 in NuRD

Nucleosome remodeling and deacetylase (NuRD) complex is a classic transcriptional corepressor consisting of a multitude of different protein assemblages that induce gene silencing and fine-tune transcription by activating class I histone deacetylases (HDACs) and directing their activity to chromatin (Bracken et al., 2019). Metastasis associated protein 1 (MTA1) is the primary and most critical interactor of RBBP4/7 within the NuRD, and this interaction is highly conserved across different organisms, at MTA1 residues 464–546 and 670–691. Both sites of MTA1 can recruit RBBP4/7 independently and are associated with the HDAC1-MTA1 dimeric complex to form HDAC1:MTA1: RBBP4/7 as the core of the NuRD complex. RBBP4 can also bind to histone H4, but due to the same interaction surface, it can no longer bind to MTA1. RBBP4 located within this complex interacts with other proteins and thus interferes with the NuRD complex to play certain epigenetic roles (Lejon et al., 2011; Millard et al., 2016; Schmidberger et al., 2016) (Figure 2B). The most classic example is the binding of friend of GATA1 (FOG1) to RBBP4. The eight positively charged residues of FOG1 bind to the negatively charged binding pocket on the surface of the RBBP4 propeller, and this interaction is highly specific in that only homologous RBBP7 can bind in the same way. As a cofactor of the erythropoietic regulator GATA1, the main function of FOG1 is to mediate the indirect binding of GATA1 to NuRD, and thus activate the expression of erythroid and megakaryocytic genes with the assistance of HDAC1/2 (Lejon et al., 2011; Yan et al., 2021; Li D. et al., 2023). The binding of malignancy factor Sal-like 4 (SALL4) to RBBP4 is similar in that the large and shallow acidic interaction surface of RBBP4 binds to 5 basic residues of SALL4, resulting in the interaction of SALL4 with NuRD and subsequent repression of tumor suppressor transcripts (Liu et al., 2018). In addition, the tumor suppressor factor Plant Homeodomain Finger 6 (PHF6) has been reported to be involved in the regulation of the NuRD complex by binding to the top surface of RBBP4 through its nucleolar localization sequence, acting mainly as a transcriptional repressor. This binding is highly conserved in the nucleoplasm, and thus mutations in PHF6 have been studied extensively in the field of cancer, especially leukemia; however, the effects of RBBP4 in mutated PHF6 are still less studied (Todd and Picketts, 2012; Liu et al., 2015). Another example is BMRF1, a heterologous Epstein-Barr virus (EBV) protein, likewise has the ability to bind to RBBP4 and regulate the NuRD complex. This binding is not conserved across herpesviruses, but is unique to EBV. After the binding of BMRF1 to the NuRD complex through RBBP4, NuRD is inhibited in its ability to respond to double-stranded DNA breaks (Salamun et al., 2019).

### RBBP4/7 in PRC2

Polycomb repressive complex 2 (PRC2) is a conserved chromatin complex composing a trimeric core of SUZ12, EED, and EZH1/2. This complex represses genes by achieving and maintaining the methylation of lysine 27 on histone H3 (H3K27me1/2/3). RBBP4/7 located at the periphery of the PRC2 core, forms a pair with SUZ12 within the complex, and its presence is crucial for PRC2’s interaction with chromosomes (Schmitges et al., 2011; Glancy et al., 2021). AEBP2 is a cofactor of PRC2 that interacts with RBBP4 and SUZ12 to enhance the stability of the core complex and promote the activities of PRC2. AEBP2 recognizes and interacts with RBBP4 not only through its common RRK-rich motif, but also through three zinc finger domains. The unique dual recognition structure with RBBP4 is a necessary basis of AEBP2 for the recruitment of PRC2 and the regulation of PRC2 activity (Kim et al., 2009; Sun et al., 2018) (Figure 2C). ARMC12 belongs to the armadillo protein family, which is closely associated with neuroblastoma progression. ARMC12 uses its ARM structural domain to bind to the N-terminal end of RBBP4 rather than the WD40 structural domain, thus enabling indirect interaction with PRC2. However, this interaction is not specific to PRC2, as silencing EZH2 or SUZ12 does not affect the physical interaction between ARMC12 and RBBP4. ARMC12 binding to PRC2 enhances EZH2 activity, leading to increased H3K27me3 levels and subsequent suppression of PRC2 target genes (Li et al., 2018). Under certain conditions, such as CpG island chromatin H3K27me3 upregulation, the dimerization of PRC2 is required to enhance binding. RBBP4 is indispensable for the formation of the PRC2 dimer. It can bind to three positively charged residues (K195, R196, K197) in the C2 domain of SUZ12, and bind with its own N-terminal helix to the helix of PHF19, which assists in the stabilization, enabling the assembly of two PRC2 monomers into a centrally symmetric dimer (Chen et al., 2020) (Figure 2D).

### RBBP4/7 in DREAM

The DREAM complex is highly conserved in both subunit composition and function, and its most important function in mammals is to bind to cell-cycle dependent genes in G1/S to regulate differentiation and proliferation (Walston et al., 2021). DREAM contains a MuvB core composed of LIN9, LIN37, LIN52, LIN54, RBBP4, and surrounding p130, E2F, and dimerization partner (DP) proteins (Gal et al., 2021). The three surrounding proteins form a trimer that uses p130 as a bridge to link to the core. p130 can bind to all proteins in the core in the G0/G1 phase, mostly to LIN52, but can also bind to other proteins, such as RBBP4, which are also necessary for DREAM to function properly (Litovchick et al., 2011; Kim et al., 2021) (Figure 2E). RBBP4 has an inhibitory effect on E2F, and downregulation of RBBP4 as well as upregulation of E2F is often observed in cells with cell cycle abnormalities (Kong et al., 2007; Li et al., 2015). In addition, the MuvB core containing RBBP4 in the G2/S phase can combine with B-MYB and FOXM1 to form the MMB complex to continue cell cycle regulation (Gründl et al., 2020) (Figure 2F). Currently, there is still limited research on the function of RBBP4/7 in the DREAM or MMB complex. Further studies on the properties of RBBP4/7 in both complexes are still required.

## RBBP4/7 in embryo differentiation

**FIGURE 3:**
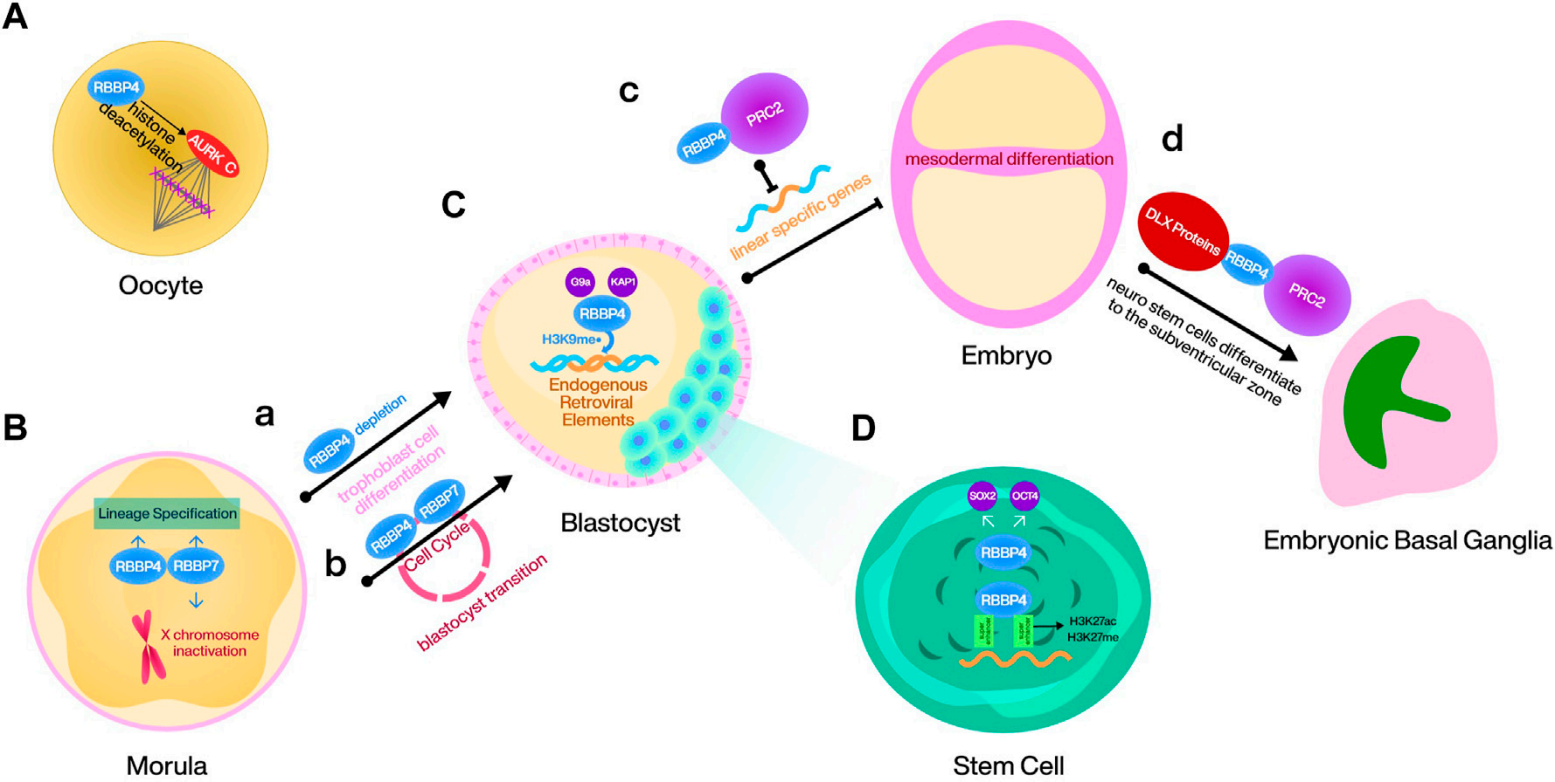
RBBP4/7 in embryo differentiation process.

As a component of epigenetic complex, RBBP4/7 regulates mouse embryo stem cell (mESC) proliferation, differentiation and the cell cycle in multiple ways. During meiosis I in mouse oocytes, RBBP4-mediated histone deacetylation plays a crucial role in regulating bipolar spindle assembly partially, through promoting the function of Aurora kinase (AURK) C. Loss of RBBP4 leads to improper histone deacetylation, causing aberrant mature spindle formation, thereby affecting the meiosis process (Balboula et al., 2015) (Figure 3A). RBBP4 recruits G9a and TRIM28 to deposit H3K9me on endogenous retroviral (ERV) elements during mouse embryonic development. Although the regulatory mechanisms induced by the silencing effect of ERV elements are unknown, RBBP4 maintains mESCs totipotency by this way (Geis and Goff, 2020; Ping et al., 2023) (Figure 3C). Additionally, RBBP4 is responsible for maintaining the levels of SOX2 and OCT4 in mESCs. Consequently, the depletion of RBBP4 is often associated with differentiation, activating abundant transposable elements that require totipotent cells (Figure 3A). This process, however, is dispensable for RBBP7 (Huang et al., 2021; Ping et al., 2023). Another study also demonstrated that RBBP4 can guide PRC2 to the loci of developmental genes and inhibit mesodermal differentiation by repressing linear specific genes (Huang et al., 2021) (Figure 3C). Moreover, RBBP4 is also a binding protein for the mESC gene super-enhancer, and its dysregulation causes reshaping of the H3K27ac and H3K27me genomic landscape (Mu et al., 2022) (Figure 3D). It has been confirmed that homozygous deletion of RBBP7 causes death in female mice. Considering that SPEN requires interaction with NuRD to initiate X chromosome inactivation and that the gene encoding RBBP7 is located on the X chromosome, RBBP7 may mediate this effect, but this conclusion needs to be confirmed later (Dossin et al., 2020; Price et al., 2022) (Figure 3B). Furthermore, the combined depletion of RBBP4 and RBBP7 in the mouse embryo during the morula to blastocyst transition leads to cell cycle disruption, disturbance of lineage specification like CDX2 and NANOG, and dysregulation of epigenetic proteins such as HDAC1/2 and histone H3.3 (Zhao et al., 2020; Xiao et al., 2022) (Figure 3B). RBBP4/7 is also essential for mouse neurodevelopment. DLX proteins, a pro-differentiation protein family in the embryonic basal ganglia, are mediated by RBBP4 to bind to NuRD and repress the regulatory elements associated with tripotent neural stem cells in the ventricular zone of the embryonic basal ganglia, thus ensuring the normal differentiation of these cells to the subventricular zone (Price et al., 2022) (Figure 3D).

## RBBP4/7 in cancer metabolism

The roles of RBBP4/7 in cancer metabolism are summarized in Table 1.

**TABLE 1:** RBBP4/7 in cancer metabolism.

Cancer type	Protein	Interactor	Mechanism	Involved effector	Functions	References
Acute Myeloid Leukemia						
	RBBP4	RNF5	RNF5 induces K29-topology polyubiquitination of RBBP4 to recruit specific gene promoters	NuRD	specific gene promoters like ANXA1, NCF1, and CDKN1A can maintain AML cells	Khateb et al. (2021)
Breast Cancer						
	RBBP4	lncRNA	RBBP4 interacts with lncRNA LCPAT1 to activate MFAP2	*N/A*	activated MFAP2 can regulate the proliferation, migration and invasion of breast cancer	Gong et al. (2020)
	RBBP4/7	BCL11A	RBBP4/7 can bind to BCL11A to recruit NuRD, PRC2 and SIN3A.	NuRD, PRC2 and SIN3A	NuRD, PRC2 and SIN3A initiate tumor-related transcriptional repression	Moody et al. (2018)
	RBBP4	BCRA1	Mutated RBBP4 allow protein relocalization of BRCA1.	*N/A*	relocated BCRA1 promote breast cancer progression	Pauty et al. (2017)
	RBBP7	*N/A*	RBBP7 is a downstream target of the tumor suppression gene WT1	*N/A*	breast cancer with high expression of RBBP7 are more susceptible to apoptosis activated by JNK signaling pathway	Li et al. (2003)
Esophageal cancer						
	RBBP7	*N/A*	*N/A*	*N/A*	RBBP7 induces cyclin-dependent kinase 4 (CDK4) expression under hypoxia and the stemness of cancer cell is upregulated	Wang et al. (2022)
	RBBP4	lncRNA	The non-coding RNA KTN1-AS1 binds to HDAC1 via RBBP4	NuRD	epithelial mesenchymal transition (EMT) is activited for tumor progression	Chen et al. (2022)
Gastric cancer						
	RBBP4	circRNA	circular RNA circ-DONSON interacts with NuRD via RBBP4	NuRD	the circular RNA circ-DONSON to promote SOX4 expression and produce tumor-promoting effects through NuRD	Ding et al. (2019)
Colorectal cancer						
	RBBP4	*N/A*	*N/A*	*N/A*	Wnt/β-catenin signaling pathway is activited by RBBP4, which leads to the dedifferentiation and EMT	Li et al. (2020)
Glioblastoma						
	RBBP4	p300	RBBP4/histone acetyltransferase p300 complex acetylates histones	Histones	the complex promotes the expression of MGMT and enhance the resistance to temozolomide	Mladek et al. (2022)
					the complex can also mediate DNA repair by upregulating Rad50 and activating the Mre11-Rad50-NBS1 (MRN) complex	Li et al. (2023b)
	RBBP4	*N/A*	RBBP4 is upregulated in gliomas by targeting of the HOXA-AS2/miR-885–5p/RBBP4 axis	*N/A*	the axis enhances the viability of malignant cells	Shou et al. (2021)
Cervical Cancer						
	RBBP4	*N/A*	*N/A*	*N/A*	the downregulation of RBBP4 can induce cervical mucosa epithelial cell carcinogenesis.	Kong et al. (2007)
	RBBP4	*N/A*	*N/A*	*N/A*	decreased RBBP4 promotes oncogene c-MYC, suppresses p53 and Rb, and decreases the expression of apoptotic genes	Zheng et al. (2013)
	RBBP4	*N/A*	*N/A*	*N/A*	RBBP4 can resist the G2/M phase arrest of cancer cells induced by irradiation.	Zheng et al. (2013)
Lung Cancer						
	RBBP4	TRIM24/28	RBBP4 together with TRIM28 and TRIM24 form a complex to promote the histone H3K9 methylation reader CBX3	*N/A*	CBX3 activates Rac1, an established effector for lung oncogenesis	Jin et al. (2022)
	RBBP4	PAF	as PAF binds to RBBP4, p130 cannot bind to RBBP4 to form a normal DREAM complex	DREAM	abnormal complex leads to abnormal transcriptional activation of DREAM target genes related to cell proliferation.	Kim et al. (2021)
Prostate Cancer						
	RBBP7	HMGB1	RBBP7 interacts directly with HMGB1	*N/A*	activited HMGB1 participates in the RNA metabolic process and thus promotes cancer cell proliferation and differentiation.	Barreiro-Alonso et al. (2021)
	RBBP7	HNF1B	RBBP7 can bind to HNF1B	*N/A*	HNF1B with RBBP7 inhibits EMT in prostate cancer via direct suppression of SLUG expression	Wang et al. (2020)
Liver Cancer						
	RBBP4	*N/A*	Low expression of RBBP4 no longer inhibits E2F1	*N/A*	increased expression of downstream genes of E2F1 such as OCT4 and SOX2 maintain cancer cell stemness.	Li et al. (2015)
	RBBP4	ZIC2	ZIC2 binds to RBBP4 to recruit the NuRF complex	NuRF	ZIC2 promotes OCT4 transcription and cancer stemness through NuRF.	Zhu et al. (2015)
	RBBP4	SALL4	SALL4 binds to RBBP4 to recruit the NuRD complex	NuRD	SALL4 silences the expression of tumor suppressors through NuRD, thereby promoting hepatocellular carcinoma cell survival.	Liu et al. (2018)

### Acute myeloid leukemia

RBBP4 is upregulated in acute myeloid leukemia (AML) and is negatively correlated with prognosis (Casas et al., 2003; Sakhinia et al., 2005). A proven mechanism is RNF5 induces K29-topology polyubiquitination of RBBP4 that promotes RBBP4 recruitment to specific gene promoters such as *ANXA1*, *NCF1*, and *CDKN1A*, and a concomitant regulation of genes implicated in AML maintenance. As RBBP4 is an important component of the HDAC complex, AML with low RBBP4 expression will be more sensitive to HDAC inhibitors, and its expression is expected to be an indicator for the use of HDAC inhibitors (Khateb et al., 2021).

### Breast cancer

RBBP4/7 is often highly expressed in breast cancer and exerts cancer-promoting effects through multiple pathways (Thakur et al., 2007; Creekmore et al., 2008). In breast cancer cells, RBBP4 interacts with lncRNA LCPAT1 to activate MFAP2, whose transcription then regulates the proliferation, migration and invasion of cancer (Gong et al., 2020). Moreover, RBBP4/7 can bind to BCL11A, a key protein that plays a tumorigenic role in triple-negative breast cancer and breast cancer stem cells, and recruit NuRD, PRC2 and SIN3A to initiate transcriptional repression. At the molecular level, the Arg-4 of BCL11A inserts into the β-propeller of RBBP4/7 at the same position where RBBP4 interacts with histone H3 (Moody et al., 2018; Choi et al., 2022). Unconventionally, mutations in the WD40 repeat sequence in the RBBP4 structure also allow protein relocalization of BRCA1, a tumor suppressor protein closely associated with breast cancer, thus promoting cancer progression (Pauty et al., 2017). However, it has also been found that high expression of RBBP7 has a cancer suppressive effect on early breast cancer cells instead, which are more susceptible to apoptosis activated by JNK signaling pathway, as RBBP7 is targeted by tumor suppression gene *WT1* to activate growth arrest- and DNA damage-inducible gene *GADD45* (Li et al., 2003).

### Gastrointestinal cancers

In esophageal cancer, RBBP7 expression is negatively correlated with patient prognosis. RBBP7 induces CDK4 expression partially mediated by HIF1α in esophageal cancer cells under hypoxia. As a stemness regulatory factor, upregulated CDK4 promotes cell viability and proliferation to promote tumor progression (Wang et al., 2022). The non-coding RNA *KTN1-AS1* binds to HDAC1 via RBBP4 in esophageal cancer to weaken the acetylation of histone H3 (ac-H3) at the promoter region of *E-cadherin*, thereby activating epithelial mesenchymal transition (EMT), an important process of tumor malignant progression (Chen et al., 2022). In gastric cancer, RBBP4 is recruited as part of the NuRF complex by the circular RNA circ-DONSON to promote *SOX4* expression and produce tumor-promoting effects (Ding et al., 2019). Similarly, colorectal cancer cells activate the Wnt/β-catenin signaling pathway via RBBP4 and may lead to the dedifferentiation and EMT (Li et al., 2020).

### Glioblastoma

The mechanism of resistance to temozolomide (TMZ) in glioblastoma has been the focus of research, and one mechanism is that drug-resistant gliomas have a highly efficient DNA repair process (Lang et al., 2021). This process is often accompanied by formation of the RBBP4/histone acetyltransferase p300 complex. The complex promotes the expression of key survival genes by acetylating histones, especially MGMT, a critical mediator of cytotoxicity for DNA alkylating agents such as TMZ (Kitange et al., 2016; Mladek et al., 2022). Alternatively, for MGMT-negative glioblastoma, the complex can also mediate DNA repair by upregulating Rad50 and activating the Mre11-Rad50-NBS1 (MRN) complex, resulting in resistance to TMZ and even radiation (Li et al., 2023b). It has been claimed that RBBP4 is upregulated in gliomas by targeting of the HOXA-AS2/miR-885–5p/RBBP4 axis, whereby the viability of malignant cells can be enhanced (Shou et al., 2021). The formation of RBBP4/p300 complexes may be one explanation for this observation.

### Cervical cancer

Unusually, RBBP4 tends to be downregulated in cervical cancer, and downregulation of RBBP4 can even induce cervical mucosa epithelial cell carcinogenesis. Decreased RBBP4 not only promotes oncogene *c*-*MYC* while suppressing *p53* and *Rb*, but also decreases the expression of apoptotic genes such as *caspase-3* and *caspase-8*. More importantly, the expression levels of the key oncogenes *E6* and *E7* in cervical cancer can increase by 8-fold and 28-fold respectively after RBBP4 knockdown. Mechanistically, the downregulation of RBBP4 results in a decrease in pRb, subsequently leading to the release of transcription factor E2F and activation of cell cycle-related gene expression (Kong et al., 2007; Zheng et al., 2013). While the positive association of RBBP4 with apoptotic proteins in cervical cancer may be related to its induction of p53-mediated apoptosis caused by estrogen deficiency (Ishimaru et al., 2006). On the other hand, different expression of RBBP4 in cervical cancer produced differences in cell proliferation after irradiation. This is because low expression of RBBP4 can resist the G2/M phase arrest of cancer cells induced by irradiation and thus become resistant to radiation therapy (Zheng et al., 2013).

### Lung cancer

RBBP4/7 are both upregulated prognostic markers in small cell lung cancer (Fukuoka et al., 2004; Wang et al., 2009). In smoking-induced lung adenocarcinoma, RBBP4 together with TRIM28 and TRIM24 form a complex to promote the histone H3K9 methylation reader CBX3, thus activate Rac1, an established effector for lung oncogenesis (Jin et al., 2022). The DREAM complex is another complex in which RBBP4 is involved in lung cancer development. Due to the binding of RBBP4 to PAF, the p130, which is supposed to bind with RBBP4, cannot form a normal DREAM complex with the MuvB core. This leads to transcriptional activation of DREAM target genes related to cell proliferation (Kim et al., 2021).

### Prostate cancer

In prostate cancer, RBBP7 interacts directly with HMGB1, which allows HMGB1 to participate in the RNA metabolic process and thus promotes cancer cell proliferation and differentiation, making the prognosis of patients with high HMGB1 expression worse (Barreiro-Alonso et al., 2021). However, RBBP7 can also bind to HNF1B as part of multiple transcriptional repressor complexes, thereby inhibiting EMT in prostate cancer via direct suppression of EMT factor SLUG expression, except that HNF1B is often repressed by EZH2 overexpression (Wang et al., 2020).

### Liver cancer

Liver tumor-initiating cells are critical for hepatocarcinogenesis, and RBBP4 is particularly critical for48 maintaining the stemness of hepatocellular carcinoma cells. A prognostic factor miRNA in hepatocellular carcinoma, miR-429, directly targets RBBP4 and reduces RBBP4 expression. Low expression of RBBP4 no longer inhibits E2F1, allowing for increased expression of downstream genes of E2F1 such as *OCT4* and *SOX2* that maintain cancer cell stemness (Li et al., 2015). Notably, ZIC2 can also recruit the NuRF complex containing RBBP4 to promote *OCT4* transcription and thus exert a similar effect (Zhu et al., 2015). However, the RBBP4 subunit can also bind to SALL4 and subsequently recruit the NuRD complex to the promoters of tumor suppressors and silence their expression, thereby promoting hepatocellular carcinoma cell survival (Liu et al., 2018).

## Therapeutic targeting of RBBP4/7

Although there has been considerable research focusing on the role of RBBP4/7 in cancer, studies on targeted therapy for RBBP4 are still very sparse, and no successful targeting of RBBP7 has been reported. This may be because RBBP4/7 functions more as a scaffold of the complex rather than as a protein directly involved in epigenetic effects, which has led people to overlook its presence. Considering the dependency of various oncogenic proteins on these complexes, interfering with their interaction with RBBP4/7 through certain methods could be a viable research and therapeutic approach.

As mentioned earlier, SALL4 in hepatocellular carcinoma cells silences tumor suppressor genes through RBBP4/7 binding to NuRD. Research has indicated that by removing certain residues and substituting three residues around the RRK motif, based on the minimal length peptide that can maintain the bioactivity of SALL4, it is possible to significantly enhance the binding capacity of this short peptide with RBBP4. The competitive inhibition of SALL4 by this engineered peptide successfully reversed the SALL4-induced gene repression (Liu et al., 2018). Similarly, the BCL11A segment composed of residues 2 to 16 can bind more tightly to RBBP4, disrupting the interaction between BCL11A and RBBP4 and reducing cellular stemness in the SUM149 cell line. This allowed researchers to elucidate the specific role of BCL11A in maintaining the activity of breast cancer stem cells. It is worth noting that the residues of this peptide segment are unmodified, and the increased binding strength is due to a 90° rotation influenced by hydrogen bonds, allowing additional binding to the lateral side of RBBP4 (Moody et al., 2018). A class of bicyclic peptide inhibitors targeting the RBBP4-MTA1 interaction has also been developed recently. Select a critical sequence on MTA1 that can bind to RBBP4 and enhance stability and affinity through bicyclization at specific residues, thereby inhibits the RBBP4-MTA1 interaction with a very low nanomolar *K*_D_ value of 8.56 nM (Hart et al., 2021). Compare with typical engineered peptides, bicyclic peptides have potential oral activity and lower immunogenicity, making them a promising direction for developing RBBP4-targeting agents. However, the challenge remains on how to effectively deliver these bicyclic peptides to the cytoplasm or nucleus of mammalian cells (Rhodes and Pei, 2017).

RBBP4 could also be pharmacologically targeted in cancer. Protopanaxadiol (PPD) is a plant active metabolite with proven anti-tumor effects in a variety of cancers (Arafa et al., 2021). Recent study indicated that PPD can bind to the lateral surface of the WD40 domain of RBBP4. The binding of PPD to RBBP4 prevents the aberrant methylation induced by PCR2 and suppresses the proliferation and migration in human colon cancer cell line HCT116 (Zhuo et al., 2022). Despite the high homology of RBBP4/7, it remains to be verified whether drugs/peptides targeting RBBP4 can also target RBBP7.

## Discussions and perspectives

Mounting evidence have shown that RBBP4/7 tends to be upregulated in cancer cells and is closely related to stemness. This is because metabolic reprogramming in cancer cells or embryo/stem cells is often accompanied by many epigenetic alterations that require a large number of transcriptional regulators, that incidentally drive the upregulation of RBBP4/7. Thus, some researchers believe that RBBP4/7 can be used as a marker for cancer (Barreiro-Alonso et al., 2021; Khateb et al., 2021). Interestingly, just as RBBP4/7 is often overlooked in the study of epigenetic complexes implicated in cancer, research on RBBP4/7 in cancer also tends to overlook the complex in which its true functional effects reside (Shou et al., 2021). That is why some studies also conclude that RBBP4/7 has an tumor suppressor effect (Li et al., 2003; Kong et al., 2007; Zheng et al., 2013). However, this does not imply that research on noncatalytic RBBP4/7 in cancer is meaningless. On the one hand, several studies indicate that RBBP4/7 is not always a component of protein complexes. It can bind to individual transcriptional coactivators such as p300 (Kitange et al., 2016; Mladek et al., 2022) and also interact with non-protein entities such as various non-coding RNAs (Li et al., 2015; Shou et al., 2021). Despite previous research indicating the involvement of non-coding RNA in epigenetic modifications, such as active miRNA mediating H3K4me3 and H3K27me3 to regulate dendritic cell differentiation in response to various immune environments (Mei et al., 2014). The research on how RBBP4/7 interacts with these non-protein entities and how RBBP4/7 exerts its function when it does not belong to protein complexes is still lacking. On the other hand, the fundamental properties of RBBP4/7 indicate that directly knocking out RBBP4/7 is not reasonable, but research on the molecular-level binding mechanisms between RBBP4/7 and its interacting partners is still scarce, limiting clear directions for targeted therapy studies and developments. Hence, studies on scaffold proteins like RBBP4/7 should emphasize the objects they are binding to and the molecular binding sites that contribute to their function. Assuming that we could develop PPI inhibitors based on the binding sites of RBBP4/7, we could further investigate the biological functions of these epigenetic complexes and potentially stimulate novel cancer-targeted therapies.

WD repeat-containing protein 5 (WDR5) is a good reference for exploring targeting RBBP4/7. WDR5 and RBBP4/7 both belong to the WD protein family. Similar to RBBP4/7, WDR5 can interact with various proteins (Wei et al., 2021) or non-coding RNAs (Huang et al., 2022) and plays a crucial role in chromatin regulation as part of complexes. However, numerous researches on WDR5 focus on its role as a bridge between the mixed lineage leukemia complex and oncoproteins like MYC (Ding et al., 2023). Hence, WDR5 demonstrates a stronger association with cancer, in contrast to the broader involvement of RBBP4/7 in various common complexes. This has also led to extensive research on drugs targeting WDR5, and some of these drugs have been validated *in vivo* (Teuscher et al., 2023). WDR5 primarily utilizes the two sides of its WD40″donut” to achieve binding to its targets (Ding et al., 2023; Teuscher et al., 2023), and most WDR5 inhibitors target the side that connects to the oncogenic protein (WDR5-interaction site) (Guarnaccia et al., 2021). Compared to the diverse and complicated binding sites of RBBP4/7, the development of WDR5 inhibitors is relatively easier. Considering the widespread presence and diverse binding sites of RBBP4/7, an inhibitor that can precisely target a specific binding site of RBBP4/7 is essential to study the specific functions of these complexes. PPD, the first small-molecule drug targeting RBBP4, represents a significant breakthrough, even though it can only target the lateral binding site of RBBP4. This is sufficient to serve as a prototype for the development of future RBBP4/7 inhibitors or even degraders.

The discovery of the lateral binding site for RBBP4 and PPD had an element of “serendipity”, but not every potential binding site could enjoy this. The difficult topologies of PPI systems are commonly deemed as “undruggable”, because small molecule drugs have difficulty forming stable structures with large and shallow protein binding surfaces, which is exactly the case for the SALL4-RBBP4 interaction (Liu et al., 2018; Wang et al., 2021). A study targeting WDR5 revealed that *in vitro* knockdown of WDR5 does not fully replicate the effects of WDR5 inhibition (Siladi et al., 2022), which illustrates the importance of specific binding sites for such scaffold proteins. Thus, engineered motif-based peptide inhibitors have become a better alternative. The two peptide inhibitors mentioned above are designed based on binding interface sequences between PPIs to enhance their affinity, which is also referred to as rational structure-based design (Wang et al., 2021). Rational structure-based design plays a very positive role in targeting proteins with well-defined structural features, such as WDR5 and HDAC1. HDAC1 has three well-defined binding motifs: a zinc-ion binding group (ZBG), a hydrophobic linker group, and a cap that binds to the enzyme. Inhibitors targeting the zinc-binding group are the most extensively studied, and some have been approved by FDA. In recent years, with the discovery of new ZBG structures and caps, a new batch of inhibitors is currently under evaluation (Su et al., 2021). As of now, in addition to the RRK motif on the WD40 domain, the binding side on the lateral of RBBP4/7 may also be a potential breakthrough point. Given the multitude of drugs targeting the WD40 protein family, computational algorithms can be utilized to quantify the geometric and chemical similarities of the binding pockets on such scaffold proteins. This approach allows for the screening of drugs among those targeting known molecular targets that may potentially interact with RBBP4/7 (Naderi et al., 2019). Additionally, with the fast evolution of protein prediction artificial intelligence models such as AlphaFold2, we can also predict more relevant conformational states and multidomain structures of RBBP/7, and the structure of RBBP4/7−Protein/RNA/DNA complexes, which can be used to guide drug development (Schauperl and Denny, 2022).

In general, RBBP4 is a widely studied but not deeply explored protein, while RBBP7 remains under-researched. However, as part of the epigenetic complex, their roles in regulating cellular differentiation, gene modification and transcription deserve greater attention. With the rapid development of computational biology and artificial intelligence, we can more conveniently study the interaction of RBBP4/7 and predict specific inhibitors to facilitate the understanding of how this multifunctional protein exerts its diverse physiological effects.

## Figures and Tables

**Table:** 

**AEBP2**	AE Binding Protein 2
**AML**	acute myeloid leukemia
**ANXA1**	Annexin A1
**ARMC12**	Armadillo repeat containing 12
**AURK**	Aurora kinase
**BCL11A**	B-cell lymphoma/leukemia 11A
**BCRA1**	Breast cancer type 1
**CAF1**	chromatin assembly factor 1
**CBX3**	Chromobox protein homolog 3
**CDK4**	cyclin-dependent kinase 4
**CDKN1A**	cyclin dependent kinase inhibitor 1A
**CDX2**	Caudal-type homeobox 2
**CHAF1**	chromatin assembly factor 1 subunit
**DLX1**	Distal-Less Homeobox 1
**DP**	dimerization partner
**DREAM**	the dimerization partner, RB-like, E2F and multi-vulval class B complex
**EBV**	Epstein-Barr virus
**EED**	embryonic ectoderm development
**EMT**	epithelial mesenchymal transition
**ERV**	endogenous retroviral elements
**EZH1/2**	enhancer of zeste homolog 1/2
**FOG1**	friend of GATA 1
**FOXM1**	forkhead box protein M1
**G9a**	Euchromatic histone-lysine N-methyltransferase 2
**GADD45**	Growth Arrest and DNA Damage-inducible 45
**GATA1**	GATA Binding Protein 1
**H3K27me**	methylation of lysine 27 on histone H3
**HDAC**	histone deacetylase
**HIF1α**	Hypoxia-Inducible Factor 1 Alpha
**HMGB1**	High mobility group box 1
**HNF1B**	HNF1 Homeobox B
**HPV**	human papillomavirus
**JARID1A**	AT-rich interaction domain 1A
**JARID1A**	Jumonji/ARID domain-containing protein 1A
**KDM5A**	Lysine-specific demethylase 5A
**mESC**	mouse embryo stem cell
**MFAP2**	microfibril associated protein 2
**MGMT**	Methylguanine methyltransferase
**MRN**	Mre11-Rad50-NBS1 complex
**MSI1**	musashi RNA binding protein 1
**MTA1**	Metastasis associated protein 1
**NCF1**	Neutrophil Cytosolic Factor 1
**NuRD**	Nucleosome remodeling and deacetylase
**NuRF**	Nucleosome Remodeling Factor
**OCT4**	Octamer-binding transcription factor 4
**PACT**	p53-associated cellular protein-testes derived
**PCNA**	Proliferating cell nuclear antigen
**PHF19**	PHD Finger Protein 19
**PHF6**	Plant Homeodomain Finger 6
**PPD**	Protopanaxadiol
**PPI**	protein-protein interactions
**pRb**	retinoblastoma protein
**PRC2**	Polycomb repressive complex 2
**RbAp**	retinoblastoma associated protein
**RBBP**	Retinoblastoma-binding protein
**RNF5**	RING finger protein 5
**RRK**	Arg-Arg-Lys
**SALL4**	Sal-like 4
**SOX2**	SRY-box 2
**SOX4**	SRY-Box Transcription Factor 4
**SPEN**	spen family transcription repressor
**SUZ12**	suppressor of zeste 12 homolog
**SV40**	simian vacuolating virus 40
**TMZ**	temozolomide
**TRIM24**	Tripartite motif-containing 24
**TRIM28**	Tripartite motif-containing 28
**WDR**	WD40 repeat
**WDR5**	WD repeat-containing protein 5
**WT1**	Wilms tumor protein
**ZBG**	zinc-ion binding group
